# Comparable outcome for autografts and allografts in primary medial patellofemoral ligament reconstruction for patellofemoral instability: systematic review and meta-analysis

**DOI:** 10.1007/s00167-021-06569-w

**Published:** 2021-04-16

**Authors:** Filippo Migliorini, Andromahi Trivellas, Jörg Eschweiler, Matthias Knobe, Markus Tingart, Nicola Maffulli

**Affiliations:** 1 Department of Orthopaedics, University Clinic Aachen, RWTH Aachen University Clinic, Pauwelsstraße 30, 52074 Aachen, Germany; 2 Department of Orthopaedics, David Geffen School of Medicine at UCLA, Los Angeles, CA USA; 3 Department of Orthopedics and Trauma Surgery, Lucerne Cantonal Hospital, Lucerne, Switzerland; 4 Department of Medicine, Surgery and Dentistry, University of Salerno, Via S. Allende, 84081 Baronissi, SA Italy; 5 School of Pharmacy and Bioengineering, Keele University School of Medicine, Thornburrow Drive, Stoke on Trent, England UK; 6 Barts and the London School of Medicine and Dentistry, Centre for Sports and Exercise Medicine, Mile End Hospital, Queen Mary University of London, 275 Bancroft Road, London, E1 4DG England UK

**Keywords:** Patellofemoral instability, MPFL reconstruction, Allograft

## Abstract

**Purpose:**

This study updates the current evidence on the role of allografts versus autografts for medial patellofemoral ligament (MPFL) reconstruction in patients with patellofemoral instability.

**Methods:**

The study was performed according to the PRISMA guidelines. In March 2021, a literature search in the main online databases was performed. Studies reporting quantitative data concerning primary MPFL reconstruction using an allograft were considered for inclusion. The Coleman Methodology Score was used to assess the methodological quality of the selected articles.

**Results:**

Data from 12 studies (474 procedures) were retrieved. The mean follow-up was 42.2 (15–78.5) months. The mean age was 21.1 ± 6.2 years. 64.9% (285 of 439) of patients were female. At the last follow-up, the Tegner (*p* < 0.0001), Kujala (*p* = 0.002) and the Lysholm (*p* < 0.0001) scores were minimally greater in the autografts. The similarity was found in the rate of persistent instability sensation and revision. The allograft group evidenced a lower rate of re-dislocations (*p* = 0.003).

**Conclusion:**

Allografts may represent a feasible alternative to traditional autograft for MPFL reconstruction in selected patients with patellofemoral instability. Allograft tendons yielded similar PROMs, rates of persistent instability, and revision. Allograft reconstructions tended to have modestly lower re-dislocation rates.

**Level of evidence:**

IV.

## Introduction

Patellofemoral instability is common, especially in young and adolescent patients [40]. The condition is multifactorial and can be associated with valgus deformity of the knee, mal-alignment syndromes, patella alta, femoral anteversion, patellar dysplasia, trochlear dysplasia, and other less common pathoanatomical conditions [4, 47, 50]. Independent from the specific cause of the instability, following patellar dislocations the medial patellofemoral ligament (MPFL) is most often damaged [10, 31]. This ligament is an important dynamic restraint to patellar lateralization during the first degrees of knee flexion, and therefore its reconstruction is often indicated when it is damaged [16, 38]. Reconstruction of the MPFL, in combination with additional stabilization procedures when indicated, yields predictable improvements in patellar stability and patient satisfaction [3, 39]. Importantly, the net forces on the patella result in a lateral vector pull on the patella in patients with recurrent patellofemoral instability [41]. The rate of re-dislocations after isolated MPFL reconstruction is 2.7–3.8% [5, 37, 52]. Graft choice is crucial to prevent surgical failures. Semitendinosus and gracilis autografts are the most common autografts used for MPFL reconstruction [28, 41]. Alternatively, synthetic grafts, allografts, or autografts such as quadriceps, patellar, adductor magnus tendons and other less commonly used grafts can be harvested for reconstruction [3, 30, 33, 42]. The role of allografts for MPFL reconstruction is still unclear. Whether allografts have comparable or even better outcomes than autografts is controversial [36, 45, 59]. Several recent clinical studies have not yet been previously considered for analysis elsewhere [13, 15, 18, 20, 27, 32, 34, 35]. Therefore, a systematic review and meta-analysis were conducted to update the current evidence and systematically compares allografts versus autografts for MPFL reconstruction in patients with patellofemoral instability. The focus of the present investigation was on patient-reported outcome measures (PROMs) and complications. A hypothesis was made that allografts and autografts achieve similar outcome following reconstruction of the MPFL.

## Materials and methods

### Search strategy

The present study was conducted according to the Preferred Reporting Items for Systematic Reviews and Meta-Analyses: the PRISMA statement [44]. The literature search was developed according to the PICO framework:

- P (Population): patellofemoral instability;
- I (Intervention): MPFL reconstruction;
- C (Comparison): autograft versus allograft;
- (Outcomes): PROMs and complications.

### Literature search

Two independent reviewers (**;**) performed the literature search in March 2021. The main online databases were accessed: MEDLINE, EMBASE, Google Scholar, Scopus, Cochrane Library. The following keywords were used in combination: knee, patella, kneecap, patellofemoral, instability, dislocation, recurrent, medial patellofemoral ligament, MPFL, reconstruction, graft, allograft, surgery, treatment, therapy, Kujala, Tegner, Lysholm, persistent, sensation, revision, re-operation, failure, re-dislocation, recurrence. The resulting titles were screened for inclusion. If the title matched the topic, the abstract was accessed. If the abstract matched the topic, the full-text article was accessed. The bibliographies of the full-text articles were screened to uncover papers not retrieved in the search process. Disagreements between the reviewers were discussed and resolved by a third author (**).

### Eligibility criteria

All the studies reporting data concerning primary MPFL reconstruction via allograft were considered for inclusion. Given the authors’ language capabilities, articles in English, Italian, German, Spanish, and French were reviewed. Level of evidences I to V, according to the Oxford Centre of Evidenced-Based Medicine [21], were eligible. Articles regarding revision settings were excluded. Articles reporting on combined and isolated procedures were included. Both recurrent and acute dislocations were considered for inclusion. Cadaveric, animal, and biomechanical studies were excluded. Revisions, registries, letters, expert opinions, commentaries, and technique guides were excluded. Only articles reporting quantitative data on PROMs and complications were included.

### Data extraction

Two authors (**;**) independently performed data extraction. Study generalities and patient baseline data (number of patients and procedures, mean age and gender), duration of follow-up, type of instability (recurrent, acute), and intervention (isolated, combined) were collected. The following patient-reported outcome measures (PROMs) were collected: Kujala Anterior Knee Pain Scale [26], Lysholm Knee Scoring Scale [58], Tegner Activity Scale [8]. Data concerning the following complications were retrieved: the persistent sensation of instability, revision and re-dislocation. Sensation of persistent instability was defined as recurrence and/or subjective sensation of subluxation or instability [46].

### Methodological quality assessment

For the methodological quality assessment, the Coleman Methodology Score (CMS) was calculated [11]. This score is divided into “part A” (analyzing the study size, follow-up, surgical approach, type of analysis, description of diagnosis, surgical technique, and postoperative rehabilitation) and “part B” (examining the outcomes criteria and related assessing procedures and the description of the subject selection process). The CMS scored the quality of the study from 0 (poor) to 100 (excellent).

### Statistical analysis

The statistical analysis was performed by the main author (**). The Shapiro–Wilk test was performed to investigate data distribution. For normal data, the mean and standard deviation were calculated. For non-parametric data, median and interquartile range were calculated. Respectively, the Student *t* and Mann–Whitney *U* tests were performed, with values of *p* < 0.05 considered statistically significant. The odd ratio (OR) effect measure was used to investigate the rate of complications, with values of $\chi^{2}$ test < 0.05 considered statistically significant. Studies that directly compared allografts versus autografts were included in the meta-analysis. The meta-analyses were performed using the Review Manager Software 5.3 (The Nordic Cochrane Collaboration, Copenhagen) for the meta-analyses. For baseline comparability, the unpaired *t* test was performed, with values of *p* > 0.5 being considered satisfactory. To evaluate the Kujala score, the inverse variance was adopted for continuous variables, with MD effect measure. Re-dislocations were evaluated through a Mantel–Haenszel analysis, with the OR effect measure. Heterogeneity was assessed through the Higgins-I^2^ test. If *I*^2^ test > 50%, high heterogeneity was detected. The comparisons were performed with a fixed model effect. In cases of heterogeneity, a random model effect was used. The confidence intervals (CI) were set at 95% in all comparisons. The overall effect was considered statistically significant if *p* < 0.05. The funnel plot of the most commonly reported outcome was performed to assess the risk of publication bias. Egger’s linear regression was performed through the STATA MP Software version 16 (StataCorp, College Station, USA) to assess funnel plot asymmetry, with values of *p* > 0.05 considered satisfactory.

## Results

### Search result

**Fig. 1:**
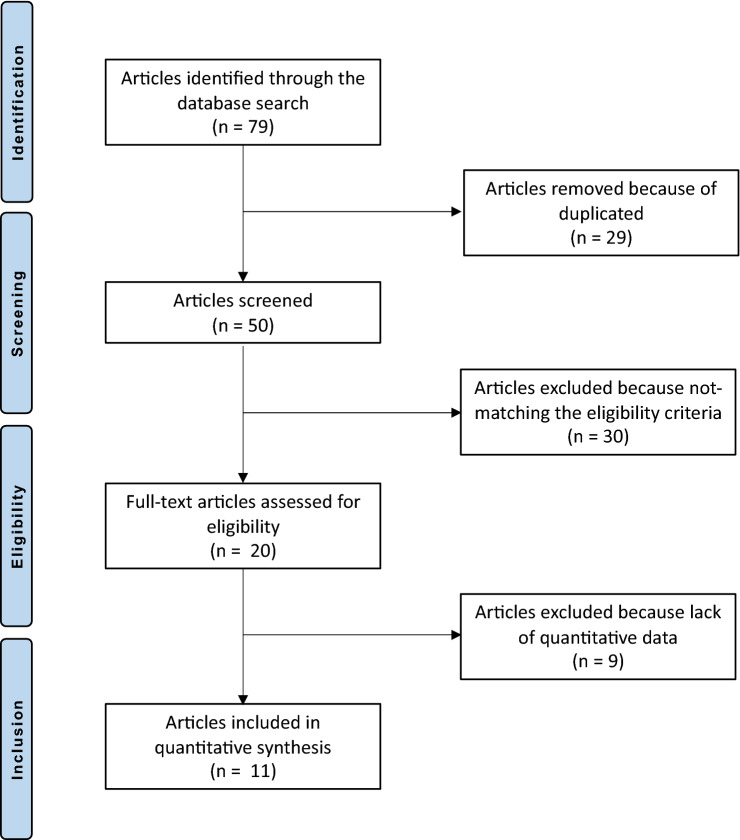
PRISMA flow-chart of the literature search

The initial search resulted in 71 published articles. Of these, 29 were duplicates and, therefore, rejected. Another 21 studies were excluded as they did not match the preferred eligibility criteria: language limitation (*N* = 1), type of study (*N* = 13), cadaveric/biomechanical work (*N* = 4), or other (*N* = 3). An additional nine studies were excluded because of lack of reporting of quantitative data under the outcomes of interests. This left 12 retrospective studies for analysis. The flow-chart of the literature search is shown in Fig. 1.

### Methodological quality assessment

The retrospective nature of the included studies is an important limitation. Moreover, no included study performed a prospective analysis or provided any randomization or blinding methods. The follow-up durations and the number of included procedures were appropriate in most studies. The descriptions of the diagnoses, even if no percentages were reported, were adequate, as were the descriptions of surgical procedures and post-operative rehabilitation protocols. Ultimately, the CMS scored 56.1/100 (37–69). The relatively low score brings witness to the moderate quality of the published studies included in the present investigation. The CMS score related to each study is shown in Table 1.

**Table 1:** Generalities and Coleman Methodology Score (CMS) of the included studies

Author, year	Journal	CMS	Follow-up (months)	Treatment	Type of graft	Samples (*n*)	Procedures (*n*)	Mean age	Female (*%*)
Calco Rodriguez et al. (2015) [9]	Rev Esp Cir Ortop Traumatol	49	12	Allograft	Semitendinosus, Gracillis, Tibialis anterior and posterior, Peroneus, quadriceps	13	13	21.0	69.2
				Autograft	Semitendinoso, Gracilis	15	16	22.0	40.0
Dragoo et al. (2017) [13]	Orthop J Sports Med	41	51	Allograft	Semitendinosus, Gracilis	8	8	36.3	87.5
Flanigan et al. (2020) [15]	Knee Surg Sports Traumatol Arthrosc	53	49.2	Allograft	Semitendinosus, Gracilis	37	57	25.8	70.3
				Autograft	Semitendinosus, Gracilis	16	30	23.5	68.8
Hendawi et al. (2019) [18]	Ochsner J	37	> 6	Allograft	Gracilis	35	35	16.0	68.6
				Autograft	Gracilis	21	21	15.3	81.0
Hohn et al. (2017) [20]	Clin Orthop Rel Res	49	24	Allograft	Gracilis	25	25	16.0	72.0
Kumar et al. (2018) [27]	Orthop J Sports Med	61	37.2	Allograft	Hamstring	36	36	15.3	61.1
			68.4	Autograft	Gracils	23	23	14.9	69.6
Li et al. (2014) [30]	J Orthop Sur Res	63	78.5	Allograft	Tibialis anterioris	65	65	29.4	56.9
Marcheggiani Muccioli et al. [32]	Knee Surg Sports Traumatol Arthrosc	69	60	Allograft	Fascia lata	17	17	21.7	35.3
Matuszewski et al. (2017) [34]	Chir Narzadow Ruchu Ortop Pol	51	15	Allograft	Tensor fasciae latae	15	15	13.1	66.7
Matuszewski et al. (2018) [35]	Medicine	65	24	Allograft	Tensor fasciae latae	22	22	15.0	54.5
				Autograft	Gracilis	22	22	15.0	68.2
Slenker et al. (2013) [53]	Phys sportsmed	66	21	Allograft	Soft tissue	23	23	20.6	65.7
				Autograft	Hamstring	12	12		
Steiner et al. (2006) [56]	Am J Sports Med	69	66.5	Allograft	Patellar	5	5	27.0	64.7
				Autograft	Adductor	23	23		
				Autograft	Quadriceps	6	6		

### Risk of publication bias

**Fig. 2:**
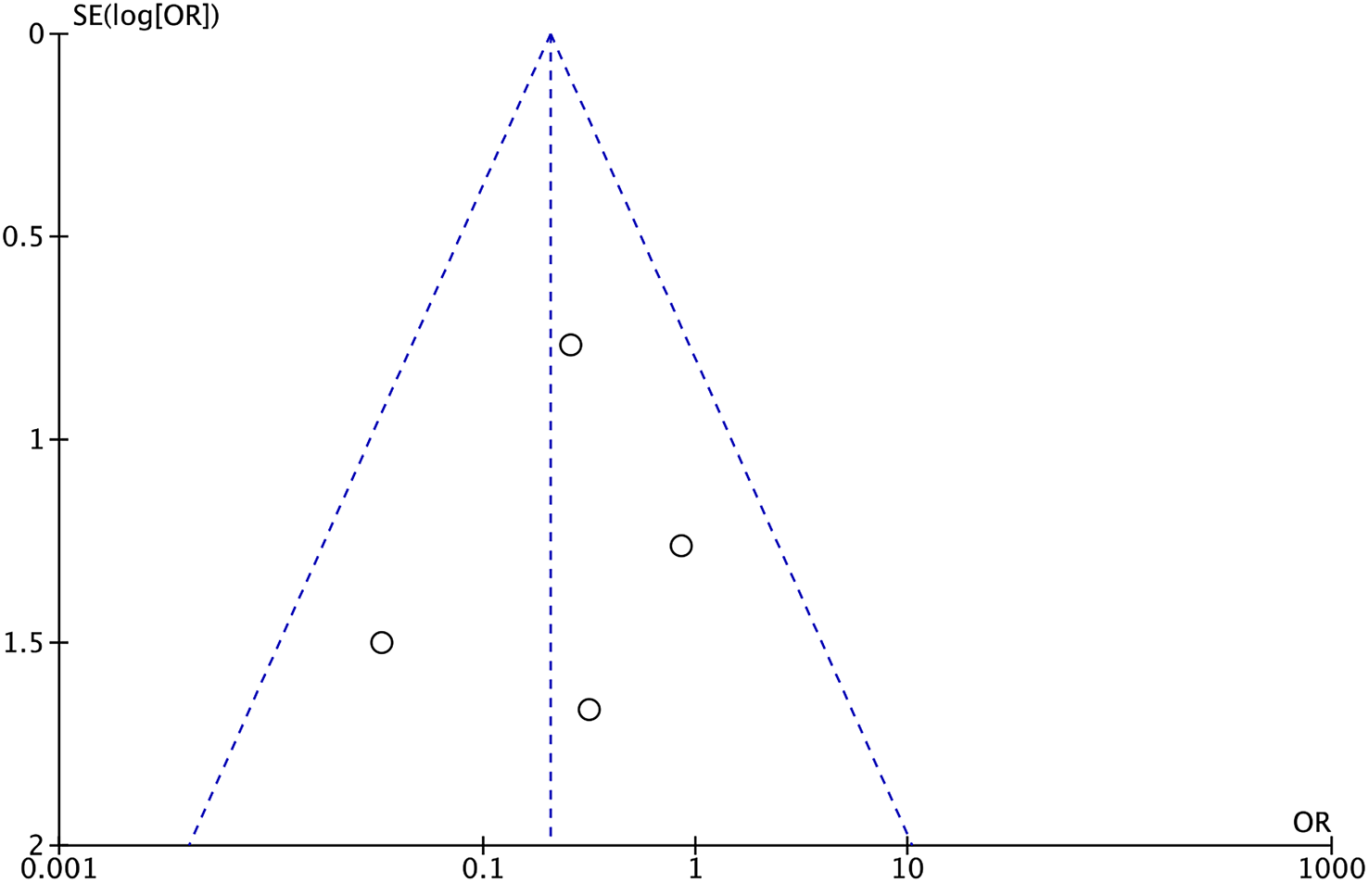
Funnel plot of the most commonly reported outcome (re-dislocation) (*SE* standard error, *OR* odd ratio)

The referral points were located into the shapes of acceptability, demonstrating good distribution. According to the Egger’s test, no statistically significant asymmetry was found. Concluding, the risk of publication bias was low. The funnel plot is shown in Fig. 2.

### Patient demographic

Data from 474 procedures (439 patients) were retrieved. The mean follow-up was 42.2 (15–78.5) months. The mean age was 21.1 ± 6.2 years. 64.9% (285 of 439) of patients were female. Good between-group comparability was found concerning patient age and gender. Study generalities and patient baseline are shown in Tables 1 and 2.

**Table 2:** Demographic data of patients

Endpoint	Allograft	Autograft	*p*
Samples	301	138	
Knees	321	153	
Mean age	21.4 ± 7.0	20.7 ± 5.1	n.s
Female	64.5% (194 of 301)	65.9% (91 of 138)	n.s

*n.s.* not significant

### Outcomes of interest

At last follow-up, the Tegner scale score was greater in the autografts (*p* < 0.0001), as were the Kujala (*p* = 0.002) and the Lysholm (*p* < 0.0001) scores (Table 3).

**Table 3:** PROMs

Endpoints	Allograft	Autograft	MD	95% CI	*p*
Tegner	4.4 ± 1.5	5.3 ± 1.7	− 0.9	− 1.203 to − 0.597	< 0.0001
Kujala	90.6 ± 3.6	91.9 ± 3.4	− 1.3	− 1.983 to − 0.617	0.002
Lysholm	83.1 ± 15.9	92.0 ± 0.4	− 8.9	− 11.428 to − 6.372	< 0.0001

Data are presented as mean and standard deviation, mean difference (MD), confidence interval (CI), and the *p* value resulting from the *t* test

Similarity was found in the rate of persistent instability sensation and revision. The allograft group evidenced a lower rate of re-dislocations (*p* = 0.003) (Table 4).

**Table 4:** Complications

Endpoint	Allograft	Autograft	95% CI	OR	*p*
Re-dislocation	2.5% (8 of 321)	9.2% (14 of 153)	0.1041 to 0.6188	0.3	0.003
Persistent instability	29.8% (17 of 57)	36.7% (11 of 30)	0.2883 to 1.8691	0.7	n.s
Revision	7.9% (3 of 38)	6.3% (1 of 16)	0.1235 to 13.3827	1.3	n.s

Data are presented as percentage, number of events and observations, odd ratio (OR), confidence interval (CI), and the *p* value resulting from the $\chi^{2}$ test

*n.s.* not significant

### Meta-analysis of direct comparisons

**Fig. 3:**
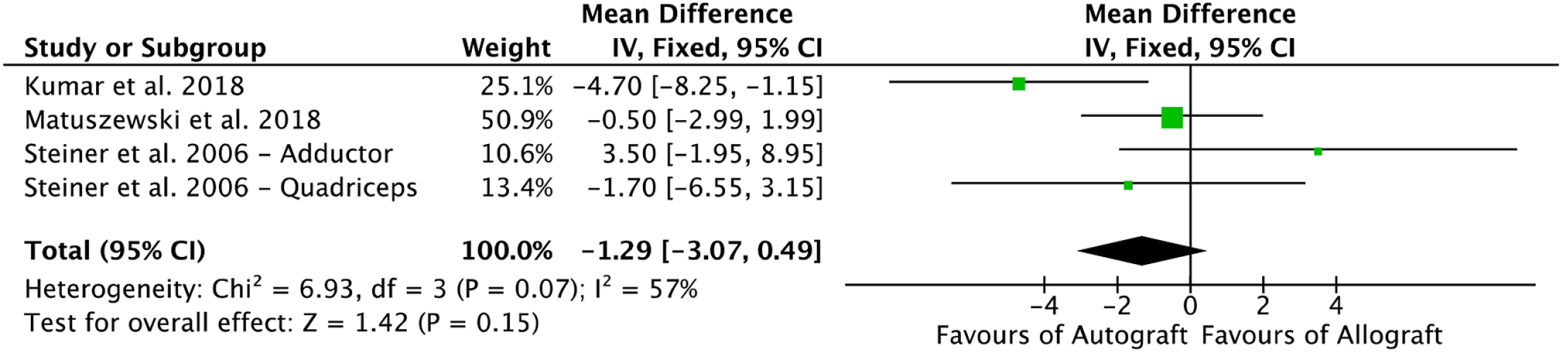
Forest plot of the outcome: Kujala score (*IV* inverse variance, *CI* confidence interval)

Three comparative studies [27, 35, 56] reported data on the Kujala score, and were included in the meta-analysis (Fig. 3). The Kujala score evidenced no difference between the two cohorts.

**Fig. 4:**
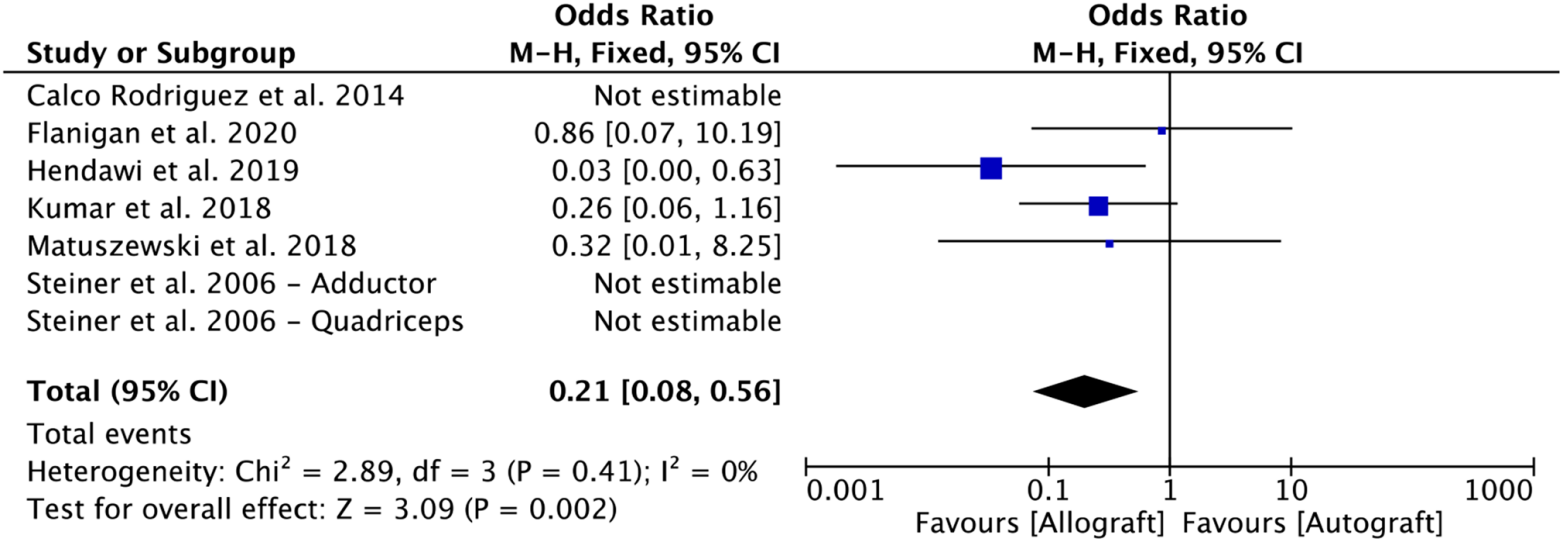
Forest plot of the outcome: re-dislocation (*CI* confidence interval)

Six comparative studies [9, 15, 18, 27, 35, 56] reported data on re-dislocation rate and were included in the meta-analysis (Fig. 4). The allograft group evidenced a lower rate of re-dislocations (*p* = 0.002).

## Discussion

According to the main findings of the present systematic review and meta-analysis, autografts and allografts may achieve similar results for MPFL reconstruction in selected patients with patellofemoral instability. Although PROMs were greater in the autografts group, they did not go above the minimal clinically important difference (MCID) [1, 8, 12, 24], and the meta-analysis did not evidence any statistically significant difference between the auto- and allografts in terms of rates of persistent instability and revision. Allograft reconstructions tended to have a lower re-dislocation rate than autografts.

Three previous systematic reviews are available. Nha et al. [45] recently performed a systematic review including only one study [13] involving eight patients for comparison into the allograft group and 21 studies on autografts. McNeilan et al. [36] performed another systematic review in 2018 analyzing two studies [30, 53] in the allograft cohort. Given the limited evidence and the poor quality of the included studies, the two studied did not allow any evidenced-based recommendations. Weinberger et al. [59] performed a systematic review including 132 allograft procedures (seven studies), concluding that autografts provided greater Kujala Anterior Knee Pain Scale scores and similar revision-rates compared to the allograft.

This systematic review and meta-analysis included the most recent evidence, increasing the number of studies and the number of outcomes of interest. In our analyses, the PROMs were similar among the two types of grafts. The intra-study variability can explain the low-moderate heterogeneity detected by the *I*^2^ and *χ*^2^ test in the Kujala score. The rates of persistent sensation of instability and revision were comparable between the two types of grafts. The analysis of re-dislocation was characterized by no heterogeneity, detecting an OR modestly in favor of the allograft group. However, only four studies were used for re-dislocation and 3 of the 4 studies have OR which crossed 1, suggesting no difference between the graft choices (Fig. 4). The result of this endpoint was strongly influenced by the studies by Hendawi et al. [18] and Kumar et al. [27]. However, their populations included only patients younger than the overall mean age of the patients in the studies that met our inclusion criteria. Hendawi et al. [18], in a retrospective study, changed from gracilis autograft to gracilis allograft because they were experiencing more failures with that autologous graft. However, instead of considering other autograft choices, such as semitendinosus, they chose to change to gracilis allografts, which incidentally had the same diameter as the gracilis autograft they were originally using [41]. They also admitted that the learning curve may have potentially biased their results. Kumar et al. [27] combined various procedures in addition to the MPFL reconstruction. In their study, seven of the allograft patients had a lateral release versus only two in the autograft patients. Their autograft cohort had six re-dislocation versus 3 in the allograft cohort. It would be difficult to conclude the re-dislocation rate is a consequence of the graft per se, as lateral release had been often used in combination with MPFL reconstruction.

Advantages of allografts are shorter surgical duration and less donor site morbidity. This may also lead to less pain and favour the early phases of rehabilitation, resulting in earlier recovery of muscle function. Potential infections and sterilization processes of allografts should be addressed briefly. Allografts carry a potential risk of disease transmission; however, sterilization processes may affect the quality and mechanical proprieties of the graft. One method of sterilization, using ethylene oxide, does not interfere with the mechanical quality of the graft but has been associated with persistent synovitis [23, 51]. To avoid this, $\gamma$-ray irradiation has been proposed. However, in vivo comparisons between irradiated and non-irradiated allografts discouraged the use of irradiated ones. Irradiated allografts were associated with a considerably higher failure rate, reducing their strength in a dose-dependent manner [49, 57]. There is a renewed interest in the prevention of infection in ligament reconstruction surgery. It is possible that devices developed for intra-articular ligament reconstruction procedures may well be used in MPFL reconstruction, especially when employing allografts [2]. Surgeons must be aware of the storage processes, sterilization methods, and standards of the tissue bank they use. Furthermore, the issue of cost-effectiveness of the allograft is still controversial. Hendawi et al. [18] concluded that allografts used for MPFL reconstructions were cheaper than autografts. The analysis was based on surgical duration, service costs, and reoperation rates. However, there are limited additional data on this specific issue. The cost-effectiveness of allografts versus autografts has been extensively investigated in other orthopaedic procedures such as anterior cruciate ligament (ACL) reconstruction. Allografts are expensive, and this has not been shown to be always offset by a shorter surgical duration [17, 43] in ACL procedures. However, even though well documented, the role of allografts in ACL reconstruction has also not been fully agreed upon [22, 25, 48, 60]. Nonetheless, comparing the use of an allograft for MPFL reconstruction with the use of allograft for ACL reconstruction may be improper because of the anatomical, physiological, and biomechanical differences between the two ligaments. Different from the MPFL, the ACL is an intraarticular structure. Intra- and extra-articular ligaments are subjected to different influences, stimulations, signaling, and vascular supply [6, 7]. Following injury, intra-articular ligaments exhibit reduced healing and higher failure rates compared to extra-articular ligaments. More specifically, when treated non-operatively, the ACL demonstrates a failure to heal rate of 90% [14]. This rate of failure is far higher than those observed in extra-articular ligaments, such as the MCL, when treated non-operatively [29]. Biomechanically, even though initially the strength of the repaired ligament is similar, over time the strength of intra-articular repaired ligaments decreases [14], while the strength of extra-articular ligament repairs increases [55].

This study has several limitations. One limitation is the small number of studies and consequently procedures available for review. Secondly, the retrospective nature of the included studies is another limitation of this work. The current literature lacks prospective analyses with blinding or sample randomization. Future studies should improve on these limitations, allowing higher-quality analyses. Additionally, inclusion and exclusion criteria of the studies included for analysis were heterogeneous. There was high variability among predisposing bone morphologies, risk factors, procedures, graft sources, follow-up, age, type of instability (recurrent, acute), and interventions (isolated, combined). This represents an important source of bias; however, considering the lack of data in the literature, no further subgroup analyses were possible. Moreover, the analyses were affected by a high level of heterogeneity; therefore, results from the present study must be interpreted with caution. Future investigations should overcome current shortcomings, performing studies with more homogeneous characteristics and indications, giving additional information concerning patient risk factors. Graft choice is complex, and to date, there is no universally agreed graft for MPFL reconstruction. Important considerations in graft choice include the ideal biomechanical proprieties (e.g., stiffness, viscoelasticity, tensile strength, thickness) of each graft. These remain undefined, and therefore no strong endorsement can be made. Future studies should also take advantage of more recently developed PROMs, such as the Banff Patella Instability Instrument (BPII) [19] and the Norwich Patellar Instability (NPI) [54] scores. These scores achieved high reliability for those patients with patellofemoral instability. Future studies with longer follow-up will be required to investigate possible attenuation of the MPFL allograft strength over time.

## Conclusion

Allografts may represent a feasible alternative to traditional autograft for MPFL reconstruction in selected patients with patellofemoral instability. Allograft tendons yielded similar PROMs, rates of persistent instability, and revision compared to autograft tendons. Patients in whom an MPFL reconstruction had been performed using allografts evidenced lower re-dislocation rate than those in whom autografts had been used.
